# Impact of bariatric surgery on cytochrome P 450 enzyme activity

**DOI:** 10.3389/fphar.2024.1372950

**Published:** 2024-03-25

**Authors:** Anna Pham, Patrick Chan, Angela Mercado, Jeffrey Wang, Zhijun Wang, Hajer Ibrahim, Hyma Gogineni, Ying Huang

**Affiliations:** ^1^ Western University of Health Sciences, School of Pharmacy, Pomona, CA, United States; ^2^ University of California, Irvine, School of Pharmacy and Pharmaceutical Sciences, Irvine, CA, United States; ^3^ Kaiser Permanente San Jose Medical Center, San Jose, CA, United States

**Keywords:** bariatric surgery, cytochrome P450, pharmacokinetics, metabolism, bypass surgery

## Abstract

Bariatric surgeries are becoming more prevalent as obesity rates continue to rise. Being that it is an effective weight-loss procedure, it can induce significant anatomical, physiological, and metabolic alterations, which affect the pharmacokinetics of various medications. Cytochrome (CYP) P450 is a group of enzymes that are primarily responsible for metabolizing most medications. Bariatric surgery may affect CYP activity and consequently alter metabolism of various medications, and the resulting weight loss may influence the metabolism of various drugs. This study investigates the impact of bariatric surgery on which CYP enzymes are affected and their effects medications. Authors of this study did an extensive literature review and research in databases including PubMed and EMBASE. The evidence was gathered for medication efficacy influenced by enzyme fluctuations to advocate for further studies for patients that undergo bariatric surgery. The search was limited to English-language results and is deemed up to date as of September 2023. There are numerous studies that indicated alterations of the CYP enzyme activity, which affects the pharmacokinetics of medications used to treat acute and chronic conditions after bariatric surgery. There are various mechanisms involved in CYP enzyme activity leading to fluctuations and the clearance of medications and subsequently compromising the efficacy and safety of these agents. It is imperative to conduct more prospective randomized control studies with longer duration to guide clinicians on how to manage medications with various CYP activity for patients’ post-bariatric surgery.

## Introduction

As the prevalence of obesity continues to rise, bariatric surgery is an increasingly popular approach to reducing bodyweight (2021, Gonzalez et al., 2021) and demonstrating a decrease in all-cause mortality (Reges et al., 2018). From 2017 to March 2020, before the halt of the National and Health Nutrition Examination Survey due to COVID-19 pandemic, it was found that among adults aged 20 and over, the age-adjusted prevalence of obesity was 41.9% (Stierman et al., 2021). In 2021, an estimated 262,893 bariatric surgeries were performed in eligible patients (morbidly obese (BMI>30 kg/m^2^) (American Society for Metabolic and Bariatric Surgery, 2022). Of these, 60% were sleeve gastrectomy which removes most of the stomach and reshapes the remainder into a sleeve and thus restricting the amount of food the stomach can hold. Furthermore, about 18% of the other procedures were gastric bypass surgery. However, bariatric surgeries induce significant anatomical, physiological, and metabolic alterations which may affect the pharmacokinetics of various medications. Cytochrome P450 (CYP) is a group of enzymes, found primarily in the liver, responsible for metabolizing medications including psychotropics, antihypertensives, and antiseizures (Ingelman-Sundberg, 2004). Along the gastrointestinal tract, enterocytes also possess CYP enzymes, expressing CYP 3A4, 2C19, and 2D6 (Paine et al., 2006).

Bariatric surgery affects CYP activity and consequently alters metabolism and the resulting weight loss may influence the metabolism of various drugs (Chen et al., 2020). Many acute and chronic condition medications display various levels of efficacy and safety even at therapeutic doses because of the fluctuation in CYP P450 enzymes activity post-bariatric surgery (Chen et al., 2020). Moreso, other enzymes such as CYP2D6, CYP1A2, and CYP2C9 have been found to show alterations.

Consequently, the pre-bariatric surgery dose utilized in managing the conditions may no longer be effective following bariatric surgery and/or induce many dose-related side-effects. Thus, this article aims summarize existing evidence regarding changes to cytochrome P450 following bariatric surgery.

There are multiple proposed mechanisms that underly the changes to CYP activity which includes:

• The recovery of CYP activity due to decreased inflammation.
• The induced-weight loss due to bariatric surgery which subsequently reduces hepatic lipid peroxidation levels and affecting cytochrome P450 protein content.

To quantify and make a correlation between drugs and enzyme activity, probe substrates are frequently employed in experimental studies. Phenotypic metrics describe the presence of the medication which is correlated with the specific enzyme. For example, midazolam is a probe substrate of CYP3A4/5. In Table 1, the inhibitors, inducers, and substrates are listed for enzymes that will be discussed in this review.

**TABLE 1:** Examples of cytochrome P450 enzymes and medications inhibitors, inducers, and substrates (Gilani and Cassagnol, 2022).

Isoenzyme	Inhibitor	Inducer	Substrate
CYP1A2 (Liver)	Amiodarone	Carbamazepine	Caffeine
	Cimetidine	Phenobarbital	Clozapine theophylline
	Ciprofloxacin	Rifampin	
	Fluvoxamine		
CYP2C9 (Liver + Intestine)	Amiodarone	Carbamazepine	Carvedilol
	Fluconazole	Phenobarbital	Celecoxib
	Fluoxetine	Phenytoin	Glipizide
	Metronidazole	Rifampin	Ibuprofen
	Ritonavir		Irbesartan
	Trimethoprim/sulfamethoxazole		Losartan
CYP2C19 (Liver)	Fluvoxamine	Carbamazepine	Omeprazole
	Isoniazid	Phenytoin	Phenobarbital
	Ritonavir	Rifampin	
CYP2D6 (Liver)	Bupropion	none	Amitriptyline
	Duloxetine		Carvedilol
	Fluoxetine		Codeine
	Paroxetine		Dextromethorphan
	Quinidine		Diltiazem
	Ritonavir		Donepezil
	Sertraline		Haloperidol
	Terbinafine		Metoprolol
			Nifedipine
			Ondansetron
			Oxycodone
			Propranolol
			Risperidone
			Tamoxifen
			Tramadol
CYP2E1 (Liver)	none	Ethanol	Acetaminophen
		Isoniazid	Theophylline
		tobacco	Verapamil
CYP3A4 (Liver + Intestine)	Amiodarone	Carbamazepine	Alprazolam
	Amitriptyline	Phenobarbital	Amlodipine
	Aprepitant	Phenytoin	Buspirone
	Carvedilol	Rifampin	Calcium channel blockers
	Chloramphenicol	St. John’s wort	Caffeine
	Cimetidine		Citalopram
	Ciprofloxacin		Clopidogrel
	Clarithromycin		Cocaine
	Codeine		Cyclosporine
	Donepezil		Diazepam
	Fluvoxamine		Erythromycin
	Haloperidol		Estradiol
	Imatinib		Lidocaine
	Ketoconazole		Losartan
	Metoprolol		Montelukast
	Paroxetine		Midazolam
	Risperidone		Quetiapine
	Ritonavir		Sertraline
	Tramadol		Sildenafil
	Verapamil		Statins
			Tacrolimus
			Warfarin zolpidem

Anatomically, drug absorption through the stomach and small intestine along with first-pass metabolism affect bioavailability of medications. As a result, the understanding of drug metabolizing enzymes is crucial to determining the importance modeling drug disposition and optimizing pharmacological treatments for patients in the bariatric surgery (Krogstad et al., 2020). This review highlights the changes in CYP enzymes following bariatric surgery and the importance of more studies required to further understand both the short- and long-term impact of bariatric surgeries on CYP enzymes.

Within the small intestine, relevant levels of cytochrome P450 enzyme that is expressed is the CYP3A subfamily which account for approximately 80% of the total intestinal P450 content. This is followed by CYP2C9, which makes up about 15%. Furthermore, the enzyme levels within the liver is significantly more than the intestine (Paine et al., 1997). CYP3A4 is the most prevalent CYP enzyme that accounts for 30%–50% of drug metabolism (Haddad et al., 2007). The alterations of CYP3A levels in the body after bariatric surgery places a patient at risk to inadequate therapy and/or dose-related side effects.

## Methods

Authors searched using PubMed and EMBASE database. The evidence was gathered for medication efficacy influenced by enzyme fluctuations to advocate for further studies for patients that undergo surgical procedures such as Roux-en-Y Gastric Bypass (RYGB), Laparoscopic Adjustable Gastric Band (LAGB), Sleeve Gastrectomy (SG), and Biliopancreatic Diversion with Duodenal Switch (BPDDS).

The search terms used included (but were not limited to): bariatric surgery, bypass surgery, RYGB, cytochrome P450, pharmacokinetics, antibiotics, anti-diabetics, antihypertensives, hypnotics, psychotropics. The search was limited to English-language results and are deemed up to date as of September 2023.

## Results

A total of 8 articles published between 2010 and 2023 including prospective cohort, simulation, and non-randomized single-group study, were used for this review. The CYP P450 enzymes studied were CYP3A4, CYP1A2, CYP2E1, CYP2C9, CYP2C8, and CYP2D6 (Bell et al., 2010; Brill et al., 2015; Brill et al., 2016; Puris et al., 2019; Rodríguez-Morató et al., 2019). CYP3A4, and CYP2B6 enzyme activity increased post-bariatric surgery (Brill et al., 2016; Puris et al., 2019; Rodríguez-Morató et al., 2019; Chen et al., 2020). In contrast, CYP2C8, CYP2C9, and CYP2D6 enzyme activity decreased post-bariatric surgery (Puris et al., 2019; Rodríguez-Morató et al., 2019). Conflicting results were reported for CYP1A2 and CYP2E1 (Bell et al., 2010; Puris et al., 2019; Rodríguez-Morató et al., 2019) [5,6,7]. In obese patients suffering from non-alcoholic fatty liver disease (NAFLD), CYP3A4 activity did not change following bariatric surgery. The results of these findings can be found in Table 2. The proposed mechanisms that underly the changes to CYP activity included recovery of CYP activity due to reduced inflammation and the induced weight loss due to bariatric surgery which reduces hepatic lipid peroxidation levels, thus affecting CYP P450 protein content (Bell et al., 2010).

**TABLE 2:** Summary of various CYP activity post-bariatric surgery.

Surgery type	CYP enzyme	Medication	Study follow-up	Sample size	Results	Study
RYGB	CYP3A	Midazolam	Follow-up 1 year	20	Increased CYP3A activity	Brill et al. (2015), Brill et al. (2016)
RYGB	CYP3A	Midazolam	Follow-up within 3 months	n/a	Increased CYP3A activity	Chen et al. (2020)
		Verapamil				
		Digoxin				
		Posaconazole				
RYGB	CYP1A2	Melatonin	First follow-up within 6–46 days	8	Increased CYP1A2 activity	Puris et al. (2019)
		Dextromethorphan				
	CYP2C8	Repaglinide			Decreased CYP2C8 activity	
	CYP2E1	Chlorzoxazone	Second follow-up within 261–412 days		Increased CYP2E1 activity	
	CYP2B6	Bupropion			Increased CYP2B6 activity	
	CYP3A	Midazolam			Increased CYP3A activity	
RYGB	CYP2D6	Dextromethorphan	First follow-up at 1 month	24	Decreased CYP2D6 activity	Rodríguez-Morató et al. (2019)
SG	CYP3A4	Paracetamol			Increased CYP3A activity	
	CYP2C9	Losartan	Second follow-up at 6 months		Decreased CYP2C9 activity	
	CYP1A2	Caffeine			Decreased CYP1A2 activity	
		Paracetamol				
RYGB	CYP2E1	No monitoring of medication	Follow-up at 15 ± 7 months	20	Decreased CYP2E1 activity	Bell et al. (2010)
LAGB	CYP 3A4/5				Unchanged CYP3A activity	
SG						
RYGB	CYP1A2	Caffeine	First follow-up before surgery	11	Unchanged CYP1A2 activity	Lloret-Linares et al. (2019)
	CYP2C9	Losartan	Second follow-up at 5–8 weeks after surgery		Increased CYP2C9 activity	
	CYP2C19	Omeprazole	Third follow up 25–30 weeks after surgery		Unchanged CYP2C19 activity	
	CYP2D6	Dextromethorphan			Unchanged CYP2D6 activity	
	CYP3A	Midazolam			Increased CYP3A4/5 activity	
RYGB	CYP1A2	Caffeine	First follow-up at week 0	108	Unchanged CYP1A2 activity	Kvitne et al. (2022)
	CYP 2C19	Omeprazole	Second follow-up at week 3		Increased CYP2C19 activity	
	CYP2C9	Losartan	Third follow-up at 9 weeks		Decreased CYP2C9 activity	
			Fourth follow-up at 2 years			

### Mechanism

#### Cytochrome P450 3A

The CYP3A family is the most abundant drug metabolizing isozyme in the human body, accounting for approximately 40% in the liver and 82% in the gastrointestinal tract (Shimada et al., 1994). Work by Puris et al. demonstrated that the AUC_0-6_ of midazolam and CYP3A4 probes were decreased to 74% after bariatric surgery (Puris et al., 2019). However, there was a slight increase in phenotypic metrics of CYP3A4 (1.4-fold) after LRYGB surgery (Puris et al., 2019). Similarly, Rodriguez-Morato, et al., found that there was a decrease in the metabolic ratio over time, which is the ratio of unchanged drug to metabolite, from both LSG and LRYGB (Rodríguez-Morató et al., 2019). Five-to 8-weeks post-RYGB, patients showed a significant increase in CYP 3A4/5 activity but normalized 25–30 weeks later (Lloret-Linares et al., 2019).

Moreover, CYP3A4’s metabolic ratio was reduced following bariatric surgery (*p* < 0.001). Midazolam, a substrate of CYP3A4, clearance was increased 1 year after bariatric surgery. The augmented hepatic CYP3A4 activity could be associated to the decrease in inflammation stemming from a reduction in inflammatory adipokines in the plasma of patients who underwent bariatric surgery (Brill et al., 2015; Brill et al., 2016). In morbidly obese patients, pre-RYGB hepatic protein levels of CYP34A were lower than post-RYGB (Chen et al., 2020). In contrast, pre-RYGB intestinal levels of 3A4 were higher than post-RYGB.

Although many studies have indicated that there is an upregulation in CYP3A4 activity post-bariatric surgery, one study showed that the change that was seen in their study was not significant enough to be seen as upregulation (Brill et al., 2016). In another study, morbidly obese (BMI>30 kg/m^2^) individuals had a higher metabolic ratio and thus a lower CYP3A4 activity, than the normal weight patients (*p* = 0.022) (Rodríguez-Morató et al., 2019). As a result, it indicated a connection between body mass index and CYP3A4 activity. CYP3A4 activity augmented because of the procedure (*p* < 0.001) with a decreased in CYP3A4 metabolic ratio over time in both SG and RYGB technique (Rodríguez-Morató et al., 2019). However, with SG, there was a statistically significant reduction in CYP3A4 activity that was achieved sooner at 4 weeks post-surgery and maintained at 6 months compared to LRYGB (Rodríguez-Morató et al., 2019).

Although a majority of the studies demonstrate changes in hepatic enzymes activities post bariatric surgery, one publication discussed changes to drug metabolizing enzymes in the GI tract. Both CYP3A4 and CYP3A5 are present in the entire GI tract. CYP3A4 is expressed at lower levels within the duodenum, increase in the jejunum and decreasing toward the ileum (Darwich et al., 2012). The proximal small intestine contains a high concentration of CYP3A. RYGB bypasses 75–100 cm of the proximal small intestine and thus medications have a decreased exposure to CYP3A. Using the Advance Dissolution Absorption and Metabolism (ADAM) model incorporated into the Simcyp Simulator, it was determined that the small intestine transit time was reduced by 38% and this showed that 87.5 cm of the small intestine was bypassed in the model. As a result, intestinal transit time is increased and lowering exposure of medications to gut metabolizing enzymes such as CYP3A4/5 (Darwich et al., 2012).

#### Cytochrome P450 1A

Hepatic CYP1A2 accounts for 18% of the isozyme content but is nearly non-existent in the gastrointestinal tract (Shimada et al., 1994). The activity of CYP1A2 temporarily decreased 1 month after LRYGB (*p* < 0.024) and returned to baseline levels 6 months later (Rodríguez-Morató et al., 2019). In contrast, one studies showed no changes in CYP 1A2 activity five-to 8-weeks *versus* 25–30 weeks post-RYGB (Lloret-Linares et al., 2019). Additionally, another study found that at 9 weeks, there was no difference in enzymatic activity between standard group compared with RYGB group. Regarding body mass index and its correlation to CYP1A2, it was found that there were no differences among the groups of normal weight, overweight, and morbidly obese. However, Puris et al. showed a significant increase in phenotypic metrics by 3.6-fold of hydroxy melatonin/melatonin AUC ratio of CYP1A2 (Puris et al., 2019).

#### Cytochrome P450 2E

Hepatic CYP2E1 accounts for 9% of the isozyme content but is nearly non-existent in the gastrointestinal tract (Shimada et al., 1994). There is conflicting evidence in the augmentation of CYP2E1 following bariatric surgery. The use of chlorzoxazone, which is a paradigm marker substrate for phenotyping CYP2E1, showed an increase of 60% of chlorzoxazone after LRYGB. In contrast CYP2E1 protein expression was significantly reduced by 17% following the weight loss surgery (Puris et al., 2019).

#### Cytochrome P450 2C

Patients who were morbidly obese with normal CYP2C9 phenotype that underwent LSG displayed a reduction in CYP2C9 metabolic ratio 1 months after bariatric surgery (*p* < 0.016) and returned to baseline after 6 months (Rodríguez-Morató et al., 2019). The metabolic ratios of CYP 2C9 in morbidly obese individuals were compared at baseline to 1-month post-surgery and 6 months post-surgery. At baseline, the metabolic ratio was 3.17 (1.77–4.56). At 1-month post-surgery, the metabolic ratio was 1.99 (1.45–3.03). The metabolic ratio at 6 months post-surgery was 3.53 (1.76–3.92). Five-to eight-weeks post-RYGB, patients showed a significant increase in CYP 2C9 activity but normalized 25–30 weeks later (Lloret-Linares et al., 2019). Additionally, the RYGB group exhibited a 1.4-fold higher mean losartan/LCA ratio (CYP2C9) compared to the diet group in another study. However, by week 3, the mean metabolic ratio in the RYGB group had decreased by 24% (95% CI 4.2, 52) from its baseline, with no subsequent alterations observed during the remainder of the study period.

Throughout the initial 3-week low energy diet phase, both the RYGB and diet groups saw comparable increases in the mean 5-OH-omeprazole/omeprazole ratio (CYP2C19), with the RYGB group showing a 43% increase (95% CI 16, 55) and the diet group showing a 48% increase (95% CI 22, 60). Subsequently, after RYGB, the mean metabolic ratio continued to rise, experiencing an additional 30% increase (95% CI 2.6, 43) over the course of 6 weeks, while no further changes were observed beyond the initial 6-week period.

The pharmacokinetic profile in serum and in urine of repaglinide were analyzed and the corresponding metabolite as well as its metabolic ratios were compared before surgery and with those after surgery 1 year later. The AUC_0-6_ of repaglinide, which is highly metabolized by CYP2C8, indicated that the activity of the enzyme decreased 44% after LRYGB (Puris et al., 2019). Before surgery the AUC_0-6_ was 5.58 (2.29–13.4) and after surgery, it was 2.43 (1.57–3.71).

#### Cytochrome P450 2D

CYP2D6 metabolic ratio increased 6 months following bariatric surgery compared to the baseline values. However, it did not reach statical significant among the two techniques of sleeve gastrectomy and Roux-en-Y gastric bypass (*p* = 0.058). Although not statistically significant, it was found that in comparison to with the normal-weight controls, patients who were defined as morbidly obese had a higher CYP2D6 activity (*p* = 0.035) (Rodríguez-Morató et al., 2019). In contrast, another study showed no changes in CYP 2D6 activity five-to 8-weeks *versus* 25–30 weeks post-RYGB (Lloret-Linares et al., 2019).

#### Cytochrome P450 2B

The pharmacokinetic profiles in serum and in urine of bupropion were analyzed and the metabolic ratios were compared to before surgery with a median year of 1 year. The post-surgery AUC_0-6_ of bupropion was 54 (43%–67%), showing a decrease from AUC_0-6_ of 67.9 (50.7–101) at baseline prior to surgery. That was decreased after bariatric surgery. However, from the phenotypic metric study, CYP2B6 increased by 1.3 folds after LRYGB (Puris et al., 2019).

## Discussion

As the prevalence of obesity rates continue to rise, bariatric surgery is an increasingly used approach to weight loss. While it may be an effective weight-loss technique, several studies have noted potential complications (Chen et al., 2020). The most noted issue would be the alteration in the pharmacokinetics of medications. Studies have analyzed the hepatic enzyme activities and protein expression to examine correlations to pharmacokinetic changes. Many of these studies have documented significant changes to the enzymes.

One proposed mechanism involves the reduction in inflammation. Through the accumulation of adipose tissue in obese patients, the excess of the macronutrients in the adipose tissue stimulates the release of inflammatory mediators such as tumor necrosis factor alpha (TNF alpha) and interleukin 6 (IL-6), which predisposes these patients to a pro-inflammatory state and oxidative stress. The proinflammatory markers change the liver gene expression profiles, thus leading to a downregulation of many drug metabolizing enzymes, such as CYP3A (Zanger and Schwab, 2013). It has been known that adiponectin are anti-inflammatory markers that allow for regulation of this response. However, in obese patients, the production of adiponectin is reduced (Ellulu et al., 2017).

Further studies found that patients who undergo bariatric surgery saw a recovery of hepatic CYP3A as the decrease in adipokines, which are inflammatory markers in obese patients, were decreased (Algahtani et al., 2016; Chen et al., 2020). Another proposed mechanism is the correlation between body max index (BMI) and the fluctuation of certain enzymes. The liver biopsies from non-alcoholic fatty liver disease (NAFLD) patients following bariatric surgery were analyzed (Bell et al., 2010). A reduction in BMI (body mass index) was correlated with a significant reduction in CYP2E1 protein content, an enzyme that is notable to partially metabolize acetaminophen to N-acetyl-p-benzoquinone (NAPQI). NAPQI is a toxic metabolite disrupts the chronic alcoholism induced microsomal ethanol-oxidizing system. As a result, it may show favorable results in reducing the risk of hepatotoxicity (Rodríguez-Morató et al., 2019).

With a reduction in CYP 2C9, medications such as phenytoin, frequently used for seizure control, can have reduced clearance. Patients may be at greater risk of dose-related side effects such as far-lateral nystagmus, ataxia, and decrease cognition. Adding to the complexity of phenytoin, the drug undergoes capacity-limited metabolism. Thus, a reduction in CYP2C9 enzyme following bariatric surgery may increase phenytoin levels exponentially. This has clinical implications as healthcare providers may have to draw phenytoin levels more frequently and may need dose reduction in the first month to prevent toxicity.

Due to increased activity in CYP3A4, dose adjustments of carbamazepine can pose challenges. Carbamazepine has been known to have auto-induction properties making its levels fluctuate during the first 2 weeks of initiation, thus making it more difficult to adjust doses accurately for patients. In the case of CYP3A being upregulated, the medication will have an increased clearance. Carbamazepine levels may be taken more frequently to account for the upregulation in CYP3A4. Digoxin, an anti-arrhythmic and heart failure medication commonly used in atrial fibrillation patients, is partially metabolized by CYP3A4. With an upregulation of CYP3A4 there will be an increase in digoxin clearance. Clinicians would have to draw digoxin levels to ensure that therapeutic levels (0.5–1.0 ng/mL for heart failure and 0.8–2.0 ng/mL for atrial fibrillation) are achieved and monitoring of EKG and to determine if increased dose adjustment required.

The following are some potential clinical implications:

• Plasma phenytoin levels may need to be measured more frequently and dose reduction in the first month may be needed to prevent toxicity.
• Plasma carbamazepine levels may be monitored more frequently to adjust dose due to upregulation of CYP3A4.
• Digoxin levels may be required post-surgery, with concomitant monitoring of EKG and heart rate to determine if increase dose adjustments are needed.

There are limitations to this review. The short follow-up, limited number of randomized control trials, and small sample size pose as limitations to this review. The longest duration of study was about 15 months. Another limitation to this study would include the small sample size that most of the studies had. As this procedure provides long-term results, it is crucial to conduct longer duration studies to follow the fluctuations of the enzyme activity potentially caused by bariatric surgery. Additionally, larger sample size with more clinical trials will allow for more robust evidence to understand enzyme activity impact on medications in bariatric surgery patients. Further subgroup analyses could explore differences in CYP alterations post-bariatric surgery based on patient demographics and clinical characteristics. It is also important to note that clinically, not all patients will undergo the same extent of hepatic changes which further adds to the complexity of empirically managing patients and their medication regimen post-bariatric surgery. Conditions that are more critical such as seizures and arrhythmias as mentioned before should be more closely monitored by pharmacists to ensure appropriate measures can be taken if medications pharmacokinetics are altered.

## Conclusion

The CYP P450 enzymes play a key role in drug metabolism and clearance. However, there have been numerous studies that have indicated alterations of the CYP enzyme activity, which affects the pharmacokinetics of medications used to treat acute and chronic conditions after bariatric surgery. From some studies, there are various mechanisms involved in CYP enzyme activity leading to fluctuations and the clearance of medications and subsequently compromising the efficacy and safety of these agents. Additional studies with longer duration may help guide clinicians on how to manage medications with various CYP activity for patients’ post-bariatric surgery. Bypass procedures can make significant alterations to the gastrointestinal tract that are both physical and physiological which can, in turn, affect the pharmacokinetic properties of medications. This is particularly important as patients undergoing these procedures tend to have several chronic obesity-related conditions that require them to be on medications. Pharmacokinetic studies using rat model of bariatric surgery have highlighted proposed mechanisms of potential alterations in absorption, distribution, metabolism, and excretion as well as the myriad of physiological changes that can further affect oral drug bioavailability. Further studies in understanding the impact of bariatric surgery on the cytochrome P450 enzymes will allow clinicians to better monitor patients on certain medications.
